# Indirect Interactions in the High Arctic

**DOI:** 10.1371/journal.pone.0067367

**Published:** 2013-06-24

**Authors:** Tomas Roslin, Helena Wirta, Tapani Hopkins, Bess Hardwick, Gergely Várkonyi

**Affiliations:** 1 Department of Agricultural Sciences, University of Helsinki, Helsinki, Finland; 2 Finnish Environment Institute, Natural Environment Centre, Friendship Park Research Centre, Kuhmo, Finland; Helmholtz Centre for Environmental Research - UFZ, Germany

## Abstract

Indirect interactions as mediated by higher and lower trophic levels have been advanced as key forces structuring herbivorous arthropod communities around the globe. Here, we present a first quantification of the interaction structure of a herbivore-centered food web from the High Arctic. Targeting the Lepidoptera of Northeast Greenland, we introduce generalized overlap indices as a novel tool for comparing different types of indirect interactions. First, we quantify the scope for top-down-up interactions as the probability that a herbivore attacking plant species *i* itself fed as a larva on species *j*. Second, we gauge this herbivore overlap against the potential for bottom-up-down interactions, quantified as the probability that a parasitoid attacking herbivore species *i* itself developed as a larva on species *j*. Third, we assess the impact of interactions with other food web modules, by extending the core web around the key herbivore *Sympistis nigrita* to other predator guilds (birds and spiders). We find the host specificity of both herbivores and parasitoids to be variable, with broad generalists occurring in both trophic layers. Indirect links through shared resources and through shared natural enemies both emerge as forces with a potential for shaping the herbivore community. The structure of the host-parasitoid submodule of the food web suggests scope for classic apparent competition. Yet, based on predation experiments, we estimate that birds kill as many (8%) larvae of *S. nigrita* as do parasitoids (8%), and that spiders kill many more (38%). Interactions between these predator guilds may result in further complexities. Our results caution against broad generalizations from studies of limited food web modules, and show the potential for interactions within and between guilds of extended webs. They also add a data point from the northernmost insect communities on Earth, and describe the baseline structure of a food web facing imminent climate change.

## Introduction

Of all animals, herbivorous arthropods are perhaps the most ecologically and economically important, not only in terms of species numbers, but also basic biomass and economic impact [1]–[4]. Yet, the roles of different forces structuring arthropod communities around the world are still poorly known. To establish why some taxa are rare and others plentiful in the arthropod assemblage of a given site, it is hardly enough to examine the properties of the species *per se* – since how species interact with each other may be more important (e.g. [5]). Such questions can only be addressed at the community level, by quantifying interspecific interactions.

Studies of interspecific interactions have recently entered a new era. From a heated debate polarized on direct top-down versus bottom-up influences ([6], [7] versus [8]–[10]), the field has advanced towards a wider consensus. In this context, ecologists seem to agree that bottom-up and top-down influences may interact to affect herbivorous arthropod populations and communities [11] and that some forces may dominate in some places for some of the time [12]. Within this more versatile paradigm, the role of indirect interactions as ricocheting between trophic levels has drawn increasing interest. Such impacts may clearly travel two ways: both through natural enemies [5], [13]–[15] and through indirect interactions via plants (for reviews, see [16]–[19]). Nonetheless, the relative roles of bottom-up-down *versus* top-down-up interactions are as yet poorly known.

What complicates the comparison of structuring forces is the potential for and complexity of indirect interactions in natural food webs. While the impacts of natural enemies and plant-mediated interactions are based on widely different processes, their imprint on realized community structure may be surprisingly similar [20]. When multiple prey species are used by the same predator, an increase in the population of one prey species can prop up the densities of the shared enemy, thus intensifying predation on the other prey [14], [21], [22]. When observed at the level of herbivore populations, the result of such an indirect mechanism may actually resemble that of classic resource competition, and the phenomenon has consequently been dubbed “apparent competition” [14]. Yet the same kinds of effects may result from top-down-up influences as mediated by the host plant: when multiple herbivore species use the host plant species, an increase in the population of one herbivore species can depress the quality or quantity of the shared food resource, thus deteriorating the performance of another herbivore [23]. How, then, do we separate the imprints of host-plant mediated and predator-mediated impacts in real communities?

During the last two decades, quantitative food webs have been advanced as a promising tool for pinpointing indirect interactions [24]. By describing not only which species interact with each other, but also how frequently these interactions occur [25], quantitative food webs may be used to formulate testable hypotheses about the role of indirect interactions in structuring natural food webs ([26], but see [27]).

Given the obvious merits of quantitative food webs, many authors have lately used them to argue that indirect interactions in general – and apparent competition in particular – may be a globally important factor in structuring communities of arthropods on plants [5], [15], [26]. In this context, almost any overlap in host use detected among parasitoids has been advanced as evidence of negative interactions occurring among the hosts [28]–[30]. Still, few studies have critically tested these underlying assumptions [26], [27], and the current enthusiasm for apparent competition may be complicated by at least four considerations:

First, any critical evaluation of the potential for apparent competition will call for food webs resolved at the species level: If we want to examine the population-level consequences of shared resources or enemies, then we had better operate with populations defined as groups of conspecific individuals with a specific diet, set of predators and shared demography. Yet, the ecological literature abounds with unresolved or only partly resolved food webs –among them the still-influential first description of an Arctic food web [31]. As stressed already twenty years ago by Martinez et al [32], such webs may be simply misleading – as it makes no sense to describe the interaction structure of nodes which in themselves are poorly defined. Equally important is the resolution of realized links among potential ones. While many authors (e.g. [33], or more recently [34], [35]) have used species co-occurrence as a proxy for realized interactions, the occurrence of two species at the same site is only a necessary condition for interaction to be possible, but offers no sufficient proof that such interaction occurs [36]–[38]. Overall then, adequate resolution of both nodes and links is crucial when it comes to deducing indirect interactions from food web structure. If the nodes of a food web do not correspond to populations, and the links between them may or may not be true, how can we infer anything about resultant population dynamics?

Second, most quantitative food webs constructed to date are based on food web modules cut out of their wider context. As measuring all interactions of a full food web is next to impossible, most authors have – per necessity – focused on measuring the interactions occurring among selected species and/or selected guilds [5], [15], [39], [40]. Nonetheless, this solution comes with a caveat: if the strength of the interactions identified and quantified in the focal module of the food web are actually surpassed in importance by other interactions not quantified, inferences derived from it are unlikely to be valid [27]. To date, few empirical studies have widened their scope beyond single guilds of enemies attacking selected sets of hosts (but see [41]–[43]) – and even here, potential interactions between the enemies themselves have been left unaddressed.

Third, few published food webs include quantifications of host specificity at multiple trophic levels: While the recent literature abounds with bipartite quantifications of plant use by herbivores (e.g. [44]–[46]) and host use by parasitoids (e.g. [30], [47]), few studies combine both (but see e.g. [42], [48]–[50]). A similar imbalance is evident within the literature addressing indirect interactions mediated by the host plant. Here, interactions among insects sharing single host plant species are well documented [17], whereas the strength and frequency of indirect interactions across the herbivores of any wider flora are less known. The question is why, when the theory and tools offered by quantitative food webs allow us to compare overlap in resource use at multiple trophic levels – and when the patterns revealed will make all the difference. If each herbivore species is restricted to a narrow set of host plant species, then the resources not shared will hardly mediate any interactions. If, on the other hand, overlap in host use is extensive, then the depletion of some host plant taxon [51] or induced changes in the quality of remaining resources (cf. [16], [17]) may reverberate across the regional insect community.

Fourth, current quantifications of food web structure are disproportionately biased towards specific latitudes and towards certain arthropod guilds. In fact, most of what we know about the quantitative structure of predator-prey food webs specifically relates to host-parasitoid systems of low and temperate latitudes [5], whereas food webs from higher latitudes are critically lacking (but vertebrate-centered food webs from high latitudes, see [31], [52]–[54]). A recent arthropod food web from Svalbard (78°55'N/11°56'E) makes an important contribution by offering commendable resolution in terms of species identity, but is as yet less clear on exactly who interacts with whom at a species level [55]. As the relative strengths of different structuring forces may vary among both guilds [56] and latitudes [39], we urgently need to quantify key processes across both latitudes and taxa. Good geographic coverage is a necessity if we want to substantiate proposed claims of generality regarding the role of any specific structuring force.

In this paper, we describe what we believe to be the first fully resolved and quantified herbivore-predator food web for the High Arctic, thereby adding a critical data point for the global assessment of food web structure and the factors affecting it. In describing the web, we take a four-step approach: 1) To quantify a simple bottom-up interaction, we first quantify the impact of an abundant herbivore on a single but central plant resource. 2) To examine the more general preconditions for indirect interactions travelling top-down-up – i.e. for plant-mediated interactions among herbivores – we then measure overlap in host plant use among Arctic insect herbivores. In doing so, we expand the prior use of quantitative overlap indices. 3) To test the idea that indirect interactions mediated by shared predators might offer strong links between herbivores, we further quantify the overlap in host use among Arctic parasitoids. 4) Finally, we expand the host-parasitoid web to encompass two additional groups of Arctic predators (birds and spiders). Through this approach, we assess how our inference regarding the strength and role of indirect interactions within a single submodule of a food web may change with an extension to surrounding modules.

## Materials and Methods

### 2.1. Study Area and Target Taxa

As a food web representative of the High Arctic, we chose the plant-arthropod assemblage of Zackenberg Valley (74°30'N/21°00'W), located in the Northeast Greenland National Park [57]. This area is characterized by a High-Arctic climate, with monthly average temperatures ranging from -20° to +7°C, and an annual precipitation of 260 mm water equivalent [58]. The study area holds a large variety in physical landscape features as well as a range of terrestrial biotopes, including fell-fields, abrasion plateaus, snowbeds dominated by *Salix arctica* Pallas (Salicaceae), fens, grasslands, salt marshes, and heathlands dominated by *Cassiope tetragona* (Linnaeus) Don (Ericaceae), *Vaccinium uliginosum* Linnaeus (Ericaceae), and the hybrid of *Dryas octopetala* Linnaeus and *D. integrifolia* Vahl (Rosaceae) [59]. As a consequence of this environmental diversity, most species known from Northeast Greenland have been detected in the area [57].

The Northeast Greenland National Park is strictly protected as specified in the Executive Order no. 7 of 17 June 1992 from the Greenland Home Rule Authority concerning the National Park in North and East Greenland, as amended by Executive Order no. 16 of 5 October 1999. These rules allow collection of plants and invertebrates within the Park if resale is not intended. Nonetheless, all access to and activities at the Zackenberg Research Station are conditional on explicit approval by the Coordination Group for Greenland Ecosystem Monitoring. Annual permits were therefore applied for and obtained from this authority. No species protected by national or international treaties were sampled in this project.

Food web reconstruction was focused on the dominant arthropod herbivores in the impoverished local fauna (i.e. Lepidoptera), their host plants and their natural enemies at the larval stage. From a qualitative perspective, the local food web surrounding these herbivores differs in many important respects from its temperate and tropical counterparts. First, while ants (and birds) are dominant predators of herbivorous arthropods in many tropical forests [60]–[63], the current study area lacks ants. Instead, the main ground-dwelling predators were hypothesized to be spiders, with families Lycosidae, Dictynidae, Thomisidae and Linyphiidae encountered abundantly at Zackenberg [64]. Second, the local food web is relatively species-poor. The flora encompasses ca 170 vascular plants ([65], Lettner, C. unpublished), whereas three years of sampling have revealed a total of 20 lepidopteran species. These species are attacked by a set of 30 larval, prepupal and pupal parasitoid species representing Hymenoptera: Parasitica and Diptera: Tachinidae, including both primary and secondary parasitoids [66]. Other potential predators include a handful of bird species, mostly sandpipers (e.g. *Calidris alba* Pallas, *C. alpina* (L.), *C. canutus* (L.) and *Charadrius hiaticula* L.), a few passerines (with the snow bunting, *Plectrophenax nivalis* (L.), being the quantitatively dominant species) and long-tailed skuas (*Stercorarius longicaudus* (Vieillot)) [67].

### 2.2. Quantifications of the Food Web

#### 2.2.1. Quantification of a specific herbivore-plant interaction

To establish the frequency of a dominant herbivore-plant interaction, we measured herbivore damage by the most common species of Lepidoptera in the area, *Sympistis nigrita* (Boisduval) ssp. *zetterstedtii* (Staudinger) (Noctuidae) on its host plant avens (typically hybrids of *Dryas octopelata* and *D. integrifolia* in the Zackenberg area: [59]). The early-instar larvae of this monophagous herbivore use the pistils and stamens of the host flower as their primary food resource.

To record the damage inflicted by *S. nigrita* on its host, we inspected all flowers of avens in 22 haphazardly-selected plots of 1×1 m each (henceforth referred to as ‘square plots’), and within a 20 cm radius of 65 *S. nigrita* larvae used as bait (see below; these plots henceforth referred to as ‘other plots’). From both materials, we estimated the average percentage of flowers damaged by larvae and its standard deviation using bootstrapping (see [68]). In brief, the plots were randomly resampled (with replacement) 10^6^ times, and the mean and SD extracted from the resulting distribution. All calculations were implemented in R [69].

#### 2.2.2 Quantifications of other trophic interactions

To establish the frequency of trophic interactions among a larger set of plants, herbivores and parasitoids, lepidopteran larvae were collected during three field seasons (June–August 2009–2011). As many of the target taxa are partly cryptic, and as they use different parts of the vegetation (ranging from *Dryas* flowers to grass stems and roots), we used a combination of semi-quantitative sampling methods to cover the essentially two-dimensional tundra habitats [66]. As our primary methods, we used visual search (on and under the vegetation), live-trapping yellow pitfalls and, less extensively, sweep netting (for details, see [66]). When combined in like proportions, these methods will offer a better quantification of the presence and relative abundance of different taxa than any single method (see [66]). Nonetheless, densities quantified in this manner are clearly not comparable to traditional temperate [28], [30] and tropical ones [25], [70], as typically based on standardized amounts of foliage [29], [71] or lower but three-dimensional vegetation [30], [43]. Nor will any other quantification known to us allow for straightforward comparisons of insect densities between Arctic tundra and three-dimensional habitats. All individuals encountered were identified in the field as based on Ahola & Silvonen [72] and placed individually in vials of 110 ml for rearing.

#### 2.2.3. Host specificity of herbivores

To establish the host plant selection of herbivores, we used two types of data: direct feeding records of larvae encountered in the field, and limited feeding trials. For the previous purpose, we recorded the host plants of individuals found feeding on plants; for the latter, larvae were offered multiple host plants during rearing.

While being reared at Zackenberg, larvae of taxa proposed to be polyphagous were offered leaves and flowers of the three most abundant plants of the local heath vegetation: *Salix arctica*, *Vaccinium uliginosum* and *Dryas octopetala* x *integrifolia*. Feeding was scored every 3–5 days.

As most Arctic Lepidoptera have a multiannual development period, all rearings were transferred to a laboratory at the University of Helsinki, Finland, by the end of the field seasons. Here, they were kept under controlled conditions (14°C, 24 hours daylight, 80% relative humidity) until late autumn, and subjected to a separate set of feeding trials. In these trials, the larvae were offered locally available plant species representing a similar phylogenetic spread as the ones offered in Greenland, but partly different species: *Salix caprea* Linnaeus and *Salix phylicifolia* Linnaeus (Salicaceae), *Vaccinium uliginosum*, and *Dryas octopetala*. In addition, *Trifolium hybridum* Linnaeus (Fabaceae; a family not present in NE Greenland) was offered to larvae in Finland, as representing a phylogenetically distant plant group. Again, feeding was scored every 3–5 days, but given the difference in plant taxa offered, this data set was analyzed separately from the Greenland one. When active feeding ceased, the larvae were exposed to an artificial winter diapause lasting until the following spring and discarded from further feeding trials.

#### 2.2.4. Host specificity of parasitoids

To establish the host specificity of parasitoids, host larvae were reared until either an adult host or parasitoid emerged, or the host individual died. Dead host larvae were searched for parasitoids, and parasitoids were identified by GV (a professional expert of parasitoid wasp taxonomy and ecology). In addition, adult parasitoids and lepidopterans were caught by Malaise trapping, by sweep netting and by yellow-pan trapping to estimate the total number of species present in the area [66].

#### 2.2.5 Generalized quantitative overlap indices

To compare the scope for different types of indirect interactions, we adapted an old tool for a new purpose. In 1999, Müller *et al*. [30] introduced a concise metric of quantitative overlap in the parasitoid complement of two host species. Here, the probability that a parasitoid attacking species *i* itself developed as a larva on species *j* is given by
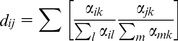
(1)where *α_ik_* is the number of parasitoids *k* found on host *i* [30]. The sums of all parasitoid individuals are included in both terms *k* and *l*, whereas the number of host individuals is summed in the term *m*. The two terms within the square bracket thus correspond to the fractions of parasitoid species *k* emerging from host species *i* and *j*, respectively. Hence, the metric *d_ij_* equals zero when host species *i* and *j* do not share any parasitoids at all, and one when host species *i* and *j* share every species in their parasitoid communities.

To characterize the potential for indirect interactions through shared host plants, we may now adopt the same metric *d_ij_* to derive a quantitative measure of overlap in host plant use among herbivores. By letting the subscripts denote the probability that a herbivore attacking plant species *i* itself fed as a larva on species *j*, we can use our data on direct feeding records to calculate a quantity which we denote the “quantitative herbivore overlap index”. This index is conceptually identical to the frequently-used “quantitative parasitoid overlap index” (e.g. [27], [30]) – but where the previous metric reflects the scope for indirect interactions mediated by a trophic level above the herbivores, the latter quantifies the same for a lower level. The unit is the same, a comparable probability.

As a routine for calculating the quantitative parasitoid overlap index is freely available in the bipartite package [73] of R [69], we adopted it for calculations and graphing. A visual representation of the full quantitative food web was derived with the same package in R version 2.13.0.

### 2.3. Expanding the Web Around a Key Herbivore

To assess how trophic links beyond parasitoids will affect our inferences regarding indirect interactions, we specifically focused on the most common species of Lepidoptera in the area, *Sympistis nigrita*. This species has a one-year life cycle, hatching from eggs in early spring, pupating after 3–4 weeks in July-August and emerging as an adult during the next summer.

**Figure 1:**
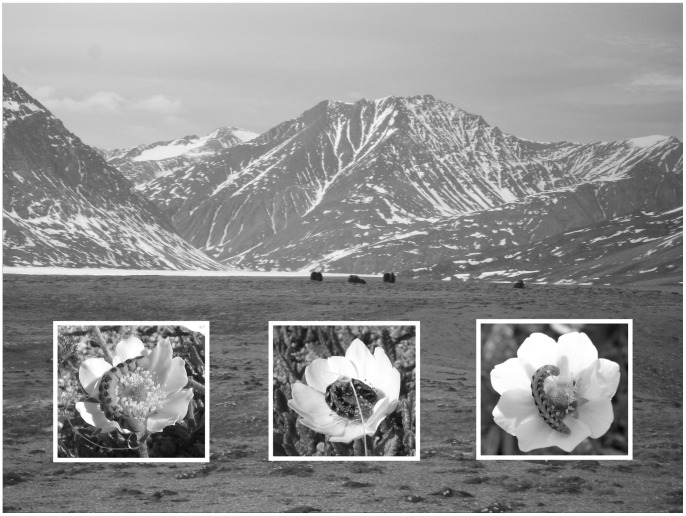
Study area and experimental design. The main picture shows the general habitat of the Zackenberg abrasion plateau, with insets illustrating larval baits used in the predation experiments. Shown from left to right is a dummy larva made of modelling clay, a live larva of *Sympistis nigrita* tethered by a thread, and a free-ranging larva for comparison.

Two trophic links were explored: predation by spiders and predation by birds. For this purpose, we used two types of baits – live and artificial larvae (Fig. 1). Both approaches involved exposing the larvae in the field as bait and checking them later for signs of attack.

Eighty-one larvae of *S. nigrita* were collected on June 20, 2011, to be used as live bait. They were reared for 5–6 days until they reached their final or next-to-final instar, then tethered to thin threads in the field (Fig. 1). All larvae were checked daily for signs of attacks. Sucked-out larvae were classified as being attacked by a spider (the only sucking true predators present in the region), while larvae missing parts were classified as being attacked by a bird. Direct observations of predators with the prey confirmed these classifications.

To supplement the laborious experiments conducted with live larvae, we used modelling clay to manufacture an additional set of 121 artificial larvae [74]. Mimicking the design used with live larvae, we tethered these baits to flowers of avens (Fig. 1). To compare attack rates on live and artificial baits, artificial larvae were first exposed simultaneously with live larvae. The artificial larvae were checked daily until July 5 and after that approximately every five days. While spiders will presumably not attack artificial bait, any larvae showing beak marks were scored as being attacked by a bird. On July 27, an additional set of 80 artificial larvae were placed on remaining patches of flowering avens.

As a measure of the strength of predation, daily attack rates (i.e. the total number of larvae attacked divided by the total number of larval days for which baits were exposed in the field) were calculated for each type of bait. To verify that artificial larvae were attacked by birds at a similar rate as live larvae, we estimated the attack rates for the time period when both types of bait were simultaneously exposed (June 25– July 11).

To compare the rate of predation by birds and spiders to that by parasitoids, we calculated – for each source of mortality – the proportion of larvae killed during the full larval period (ca 25 days; Roslin, T., Várkonyi, G. and Hardwick, B., unpublished data) based on the daily attack rates. For spider predation, we used attack rates on live bait between June 25 and July 11. For bird predation, we used data from the same time period, but combined daily attack rates on live and artificial bait. For parasitism, we used the overall data (not daily attack rate) on *S. nigrita* specimens collected between June 17 and August 12, 2011. As most parasitoid species included in these data appear to attack early-instar larvae, we used the overall fraction of larvae yielding parasitoids in 2011 as an estimate of the proportion parasitized during the larval period (*n* = 457). We converted daily predation rates (*P_d_*) to mortality from predation over the full 25 day larval period (*P*_25_) as
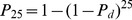
(2)


Finally, to assign a single cause of mortality to each larva, we had to account for the fact that a substantial portion of parasitized larvae are actually predated by spiders or birds before the parasitoid has had time to hatch. Making the explicit assumption that parasitized and unparasitized larvae are equally likely to be predated, the parasitism rates derived above were adjusted by subtracting predated larvae, as
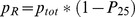
(3)where *P*_25_ is the combined bird and spider predation over the larval period, *p_tot_* the overall parasitism of *S. nigrita,* and *p_R_* is the realized parasitism rate (proportion of host larvae which produce a parasitoid). The proportions of parasitized larvae killed by birds and spiders, separately, were calculated as
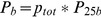
(4)where P25b is mortality caused by birds (and spiders, respectively) over the 25-day larval period, and ptot the overall parasitism of S. nigrita. The same rationale was adopted to estimate the fraction of parasitized larvae killed by spiders.

## Results

**Figure 2:**
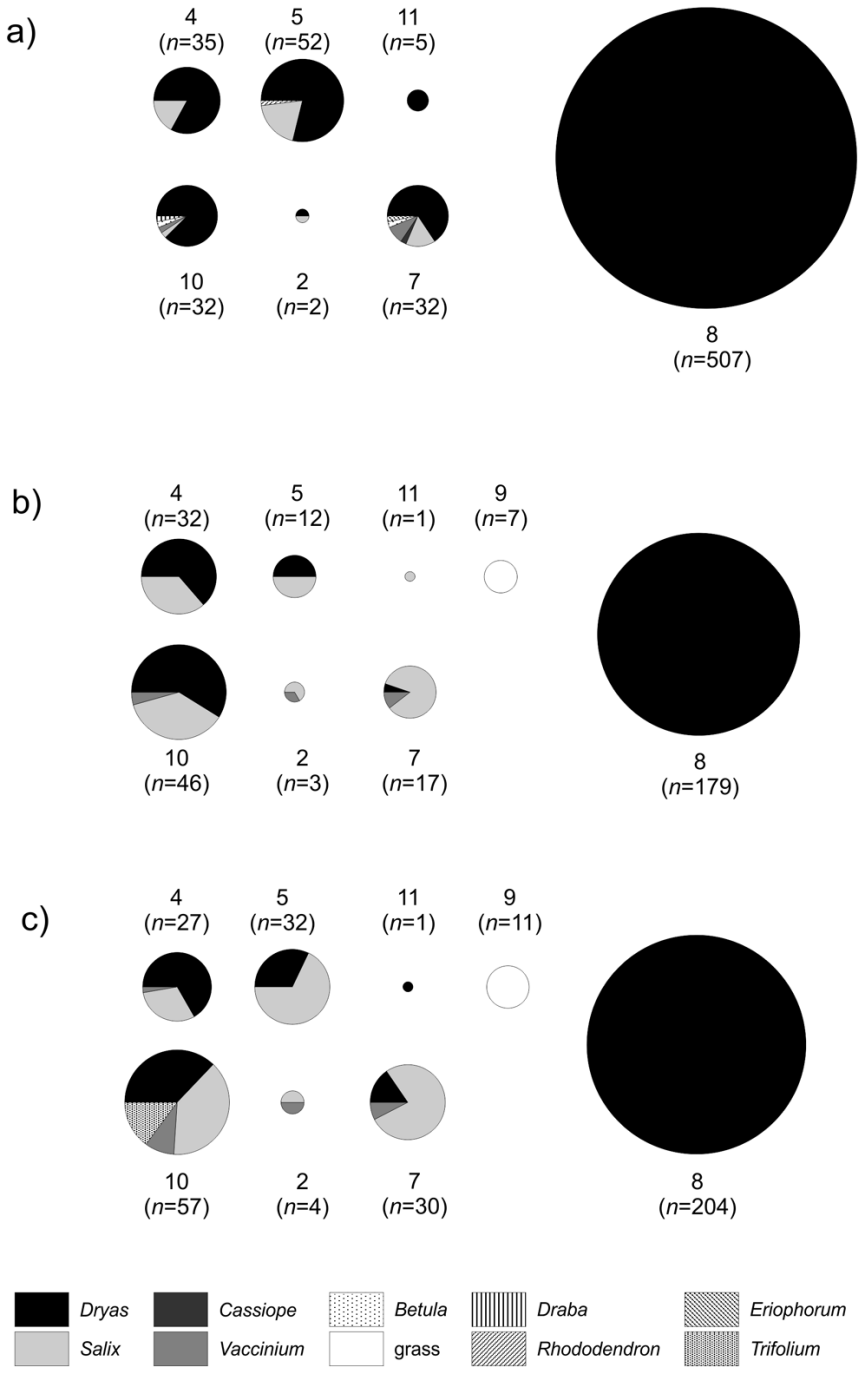
Host plants used by the Lepidoptera of the Zackenberg Valley, Northeast Greenland. A) field records of host plant use (only larvae found actively feeding are included here); B) acceptance of host plants in laboratory rearings conducted at Zackenberg; C) acceptance of host plants in laboratory rearings conducted in Helsinki, as based on locally available species (congeneric with their High-Arctic counterparts). Panel A) is based on observations from 2009–2012; panels B) and C) on data from 2009–2010. Arabic numerals refer to species as given in Table 1. [Footnote:] *Gynaephora groenlandica* here offers a special case as, contrary to other species, the larvae spend most of their time basking, not feeding [101]. Hence, we have obtained very few records of host plant selection in the field. In the laboratory, the larvae (*n*_total_ = 95) were only fed *Salix*, thereby contributing no information.

In total, we collected and reared 1420 lepidopteran larvae feeding on 9 host plant species (Fig. 2). Of these, 233 herbivore individuals (16%) were found to be parasitized by parasitoid wasps or flies. Our rearings represent 13 lepidopteran species, twelve parasitic wasp (Hymenoptera: Braconidae and Ichneumonidae) and two parasitic fly (Diptera: Tachinidae) species (Table 1; for further details on specific taxa, see [66]).

**Table 1:** Host and parasitoid taxa encountered in rearings from the Zackenberg Valley.

Trophic level	Code	Family	Species	Author & year
2	1	Lepidoptera: Pterophoridae	*Stenoptilia islandica*	(Staudinger, 1857)
2	2	Lepidoptera: Pyralidae	*Pyla fusca*	(Haworth, 1811)
2	3	Lepidoptera: Pieridae	*Colias hecla*	Lefèbvre, 1836
2	4	Lepidoptera: Nymphalidae	*Boloria* spp.^a^	
2	5	Lepidoptera: Geometridae	*Entephria* sp.^b^	
2	6	Lepidoptera: Lymantriidae	*Gynaephora groenlandica*	(Wocke, 1874)
2	7	Lepidoptera: Noctuidae	*Syngrapha parilis*	(Hübner, 1809)
2	8	Lepidoptera: Noctuidae	*Sympistis nigrita* ssp. *zetterstedtii*	(Staudinger, 1857)
2	9	Lepidoptera: Noctuidae	*Apamea zeta*	(Treitschke, 1825)
2	10	Lepidoptera: Noctuidae	*Polia richardsoni*	(Curtis, 1834)
2	11	Lepidoptera: Noctuidae	*Euxoa adumbrata* ssp. *drewseni*	(Staudinger, 1857)
3	12	Hymenoptera: Ichneumonidae	*Campoletis horstmanni*	Jussila, 1996
3	13	Hymenoptera: Ichneumonidae	*Diadegma majale*	(Gravenhorst, 1829)
3	14	Hymenoptera: Ichneumonidae	*Hyposoter frigidus*	(Lundbeck, 1897)
3	15	Hymenoptera: Ichneumonidae	*Hyposoter deichmanni*	(Nielsen, 1907)
3	16	Hymenoptera: Ichneumonidae	*Ichneumon discoensis*	Fox, 1892
3	17	Hymenoptera: Ichneumonidae	*Cryptus leechi*	Mason, 1968
4	18	Hymenoptera: Ichneumonidae	*Gelis maesticolor*	(Roman, 1933)
4	19	Hymenoptera: Ichneumonidae	*Mesochorus* sp. nec *agilis* Cresson	
3	20	Hymenoptera: Braconidae	*Microplitis lugubris*	(Ruthe, 1860)
3	21	Hymenoptera: Braconidae	*Cotesia* spp.	
3	22	Hymenoptera: Braconidae	*Hormius moniliatus*	(Nees, 1811)
3	23	Hymenoptera: Braconidae	*Dolichogenidea* sp.	
3	24	Diptera: Tachinidae	*Exorista thula*	Wood, 2002
3	25	Diptera: Tachinidae	*Peleteria aenea*	(Staeger, 1849)

Trophic levels separate hosts (trophic level 2) from parasitoids (trophic level 3) and hyperparasitoids (trophic level 4). Taxon-specific numbers in column “Code” identify taxa in Figs. 2, 4 and 5.

a As the larval characters of *Boloria chariclea* (Schneider, 1794) and *Boloria polaris* (Boisduval, 1828) are unknown, they have been combined as *Boloria* spp.

b The identity of *Entephria* taxa occurring at Zackenberg is currently being clarified by rearing and DNA sequencing techniques**.**

**Figure 3:**
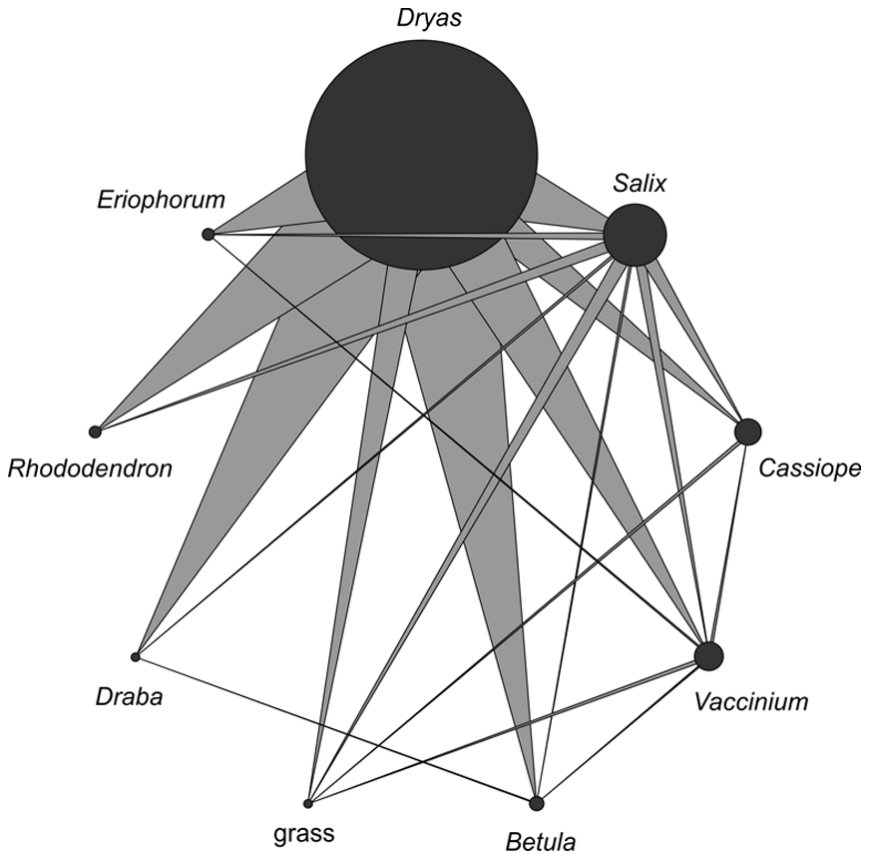
Quantitative herbivore overlap diagram showing the amount of herbivores shared between plant species. Plants are represented by discs, the size of which shows the total amount of herbivores using the respective species (as collected while feeding on it). The width of each vertex represents the amount of herbivores shared between one plant and another (i.e. the probability that a herbivore feeding on plant species *i* itself fed as a larva on species *j*).

**Figure 4:**
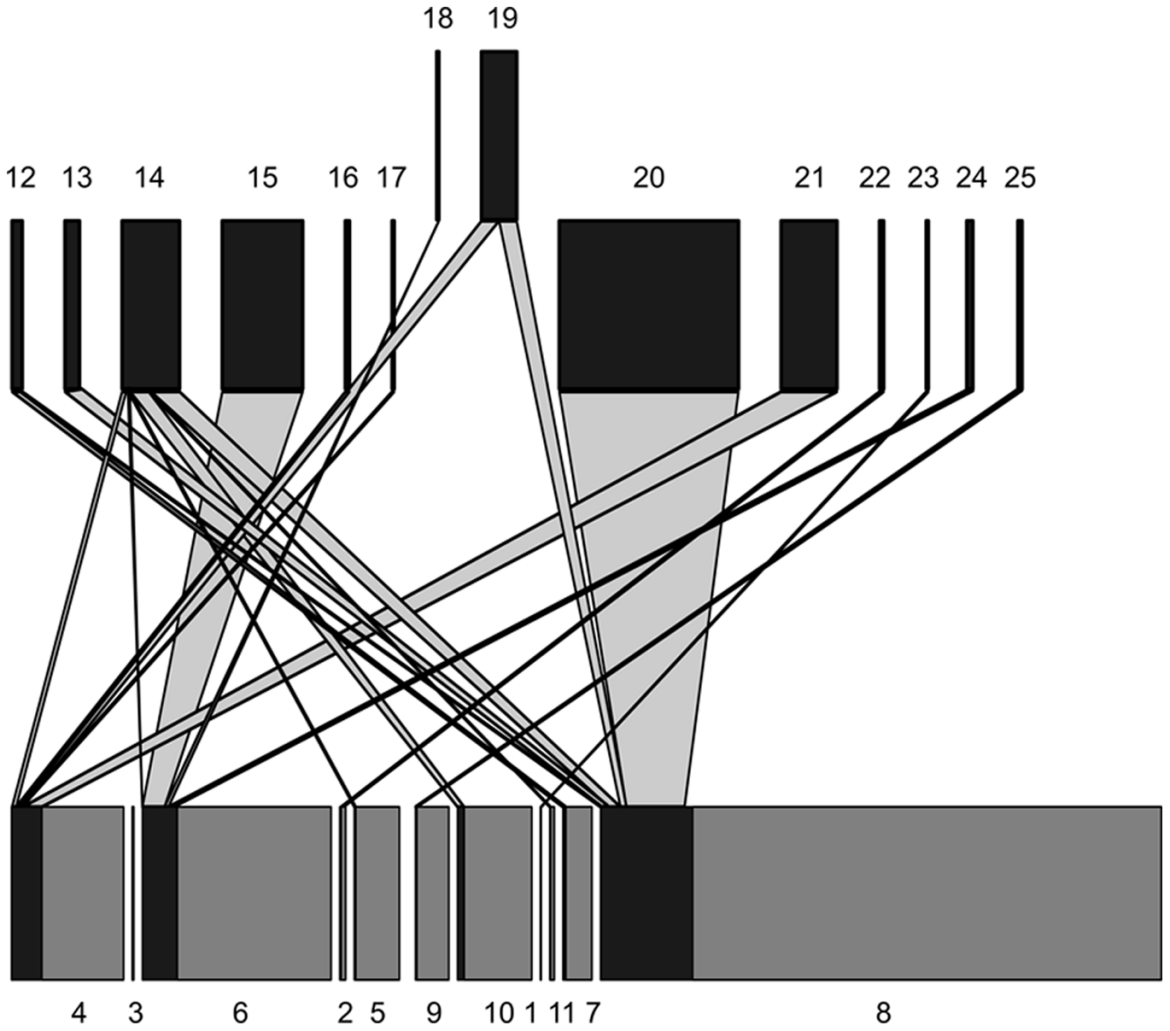
Quantitative host-parasitoid food web of Lepidoptera and their parasitoids of the Zackenberg Valley. Each bar at the lower level represents a host species and each bar at the upper level a parasitoid species. Hyperparasitoids have been offset to a higher level. Inside the host bars, the black part indicates parasitized host individuals and the grey part unparasitized ones. Lines between hosts and parasitoids describe trophic interactions, with the width of the line proportional to the frequency of the interaction. For each bar, its width represents the relative abundance of the respective taxon, with parasitoids scaled as 6.1× hosts. For more details on specific parasitoid taxa, see [66]. Arabic numerals refer to species as given in Table 1.

**Figure 5:**
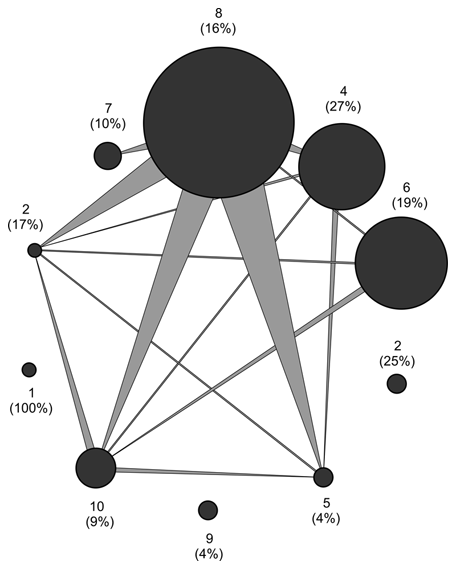
Quantitative parasitoid overlap diagram showing the amount of parasitoids shared between lepidopteran species. Host species are represented by discs the area of which shows the total amount of parasitoids developing on the respective host, and the width of each vertex represents the amount of parasitoids shared between one host and another. Percentages reported under each species identify taxon-specific parasitism rates for 2009–2011. Arabic numerals refer to species as given in Table 1.

The frequency of the dominant herbivore-plant interaction proved low: The bootstrapped average herbivory rate of *S. nigrita* on flowers of *Dryas* was 10.0% (SD 2.6%) for ‘square plots’, 8.4% (SD 2.2%) for the ‘other plots’, and 9.1% (SD 1.7%) across the two materials combined. Beyond this monophagous species, the host specificity of both herbivores and parasitoids was found to be variable, with broad generalists dominating both trophic layers (Figs 2, 3, 4, 5). Most herbivores were found feeding on the quantitatively dominant plant species of the area: *Salix arctica*, *Vaccinium uliginosum*, and *Dryas octopetala* x *integrifolia* (Fig. 2). Feeding trials confirmed that most species accepted all host plants offered, albeit in variable proportions (Fig. 2). Some species did prove strict specialists of given plant taxa, in particular the quantitatively dominant *Sympistis nigrita* (feeding exclusively on *Dryas*) and the less abundant *Apamea zeta* (feeding exclusively on grasses; Fig. 2). As the plant species represent distinctly different families, a majority of the herbivores can then be defined as broad polyphages. Of the local resource base, the highly abundant *Dryas* was frequently used by a majority of species, thereby providing a major potential for indirect interactions through the lower trophic layer (Fig. 3).

Links within the host-parasitoid part of the food web suggested scope for classic apparent competition through shared natural enemies: all but three of the host species yielding any parasitoids in the rearings also shared parasitoids with other hosts (Figs 4–5). Of specific taxa, *Sympistis nigrita* produced the most parasitoids shared with other taxa (Fig. 4).

**Figure 6:**
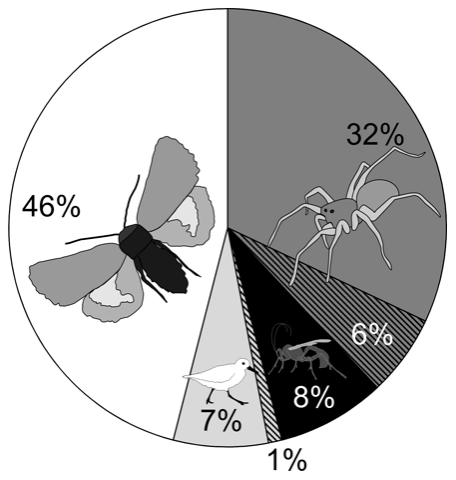
Mortality rates on*Sympistis nigrita* as incurred by different predator guilds. During the 25 days that the species spends as a larva, we estimate that 38% will be eaten by spiders, 8% will be eaten by birds and 15% will be parasitized, leaving 46% to successfully pupate. The fact that a fraction of larvae carrying parasitoids will actually be consumed by either birds or spiders (cross-hatched sections within respective fractions) yields scope for indirect interactions among predator guilds.

Overall, 15.1% of the *Sympistis nigrita* collected in 2011 (*n* = 457) were found to be parasitized, as compared to 21.5% of the larvae collected during 2009–2010 (*n* = 298; Fig. 5). When the food web around this key herbivore was expanded to include spiders and birds, we observed a daily mortality rate of 0.3% due to bird attacks (with 8 bird attacks during 2399 bait days), and 1.9% due to predation by spiders (with eleven spider attacks during 587 bait days). When translated to mortality rates over the full larval period, this implies that birds kill as many *S. nigrita* larvae (8%) as do parasitoids (8%), and that spiders kill many more (38%; Fig. 6).

Adding credence to the current results, different types of baits used to estimate predation rates by birds yielded similar results. During the time period used to compare live and artificial *S. nigrita* (June 25 to July 11), live *S. nigrita* experienced an attack rate of 0.004 bird attacks per bait and day, whereas the corresponding figure for artificial larvae was an almost identical 0.003. Hence, the rate of bird attacks on artificial and live baits did not detectably differ (Fisher’s exact test, *P*>0.99).

## Discussion

While the notion of simple Arctic food webs has been deeply engrained in ecological theory [75]–[78] [79], our web confirms a different impression emerging from recent empirical studies: that the food webs of high latitudes may be characterized by unexpectedly high species diversities, by a wealth of trophic connections, and by many stacked trophic layers [55], [80], [81]. In all, twenty species of butterflies and moths have been encountered in Zackenberg Valley, along with 27 wasp species and three species of flies parasitizing Lepidoptera [66]. Adding complexity to the parasitoid community are the hyperparasitoids, which effectively represent a further trophic level.

What should be regarded as “unexpectedly” high diversity is clearly a moot point. An explicit theory of why Arctic food webs should be simple was offered by Oksanen [79] – but here simplicity arises from a conflict between low productivity and high energy demands of endotherms. In this context, Hodkinson & Coulson [55] have stressed that Arctic food chains of ectotherms may not be constrained by the same rules. What is more, the same authors have emphasized that the myth of simple Arctic food webs may emanate from poorly resolved and endotherm-centered webs propagating through the literature for nearly a century ([31] and still being generated, though partly for other purposes [53], [54]). Where the appropriate effort has been invested in resolving the species-level structure of Arctic arthropod food webs, these webs have actually proven complex. A prime example concerns the epitome of “simple” Arctic food webs – the original “food cycle” described from Svalbard by Elton [31]. When revisited by Hodkinson & Coulson [55], the small set of compound taxa depicted here were actually resolved into an intriguing diversity of individual species and trophic layers, connected by diverse links [55].

From the relatively complex structure of the food web emerging at Zackenberg – and from the relatively high rates of parasitism and predation rates observed – biotic interactions seem as likely to contribute to shaping the local herbivore communities here as elsewhere on the globe (e.g. [82]). This observation runs contrary to the widespread and long-prevailing view that biotic interactions, including herbivory, predation and competition are more intense at low than high latitudes (e.g. [83]–[85]) – but concurs with recent studies finding no latitudinal trends in the strength of e.g. plant-herbivore interactions [86].

In terms of the specific biotic interactions structuring the herbivore community of Northeast Greenland, direct resource competition will appear unlikely, as the general level of herbivory observed in the field seems to remain uniformly low across years and host species. This impression is supported both by our quantification of the *Dryas*-*Sympistis* interaction and by less rigorous estimates of herbivory rates across the surrounding vegetation: While we have so far not combined our search for lepidopteran larvae with any strict measurement of herbivore damage, the methods involved force us to scan through several hundreds of thousands of m^2^ of foliage per summer. Our estimate from this admittedly vague but extensive survey is that the proportion of foliage consumed is typically very low (below 1%) and always low (under 10%), regardless of plant species, time (n = 4 years) and specific area (n = some 10 km^2^). While more satisfactory quantifications will follow, our summary observations suggest that direct competition through resource depletion is an unlikely source of competition among the herbivores of Zackenberg. This observation from the High Arctic contrasts with classical theories predicting strong bottom-up control in low-productive habitats [87], [88], but agrees with recent studies questioning such relationships among insects [79].

Importantly, low levels of herbivory compared to higher levels of predation (see below) offer no proof as such that top-down forces would dominate over bottom-up influences in determining herbivore densities (cf. [53], [54]). After all, changes in plant quantity may reveal little about changes in plant quality [18], [89], [90]. From the latter perspective, induced responses as mediated by a lower trophic level appear as a potential force linking herbivore taxa to each other. Contrary to classic assumptions [91], the arthropod herbivores of the Zackenberg Valley emerge as broad generalists, with most species observed feeding on (or at least accepting) the quantitatively dominant food plants. As these plant species represent different growth forms and families, the patterns found reveal no systematic signal, but true consumption of most things green. These patterns are consistent with recent findings of unexpectedly low host specificity of herbivorous arthropods around the globe [46], [92], [93], and – in principle – with a proposed decline in herbivore specificity with increasing latitude [94].

From a methodological perspective, the current patterns are affected by both host plant acceptance and host plant availability, and may then not be directly compared to quantitative host specificity as recorded elsewhere [44], [56]. What they do show is that a major fraction of local herbivore species will factually develop on the same host plant species (Fig. 3), and that any effects on local resource availability and/or quality [16], [23] may thus percolate from one species to another in the regional insect community. Importantly, the current results offer no proof of realized effects, only the potential for indirect interactions (cf. [27]). Hence, the main finding is a need for experimental studies pinpointing the net consequences of host-plant-mediated interactions among Arctic herbivores.

In terms of effects mediated by the higher trophic level, our analysis of parasitoid overlap among host species reveals some potential for apparent competition – in particular through the abundant host *S. nigrita* (Fig. 5). While similar patterns have caused multiple authors to argue for an important role of apparent competition among parasitoid species in structuring herbivore communities worldwide [15], [26], this inference was immediately challenged by the next step of our analysis: when the parasitoid-host web was expanded to added predator guilds, and different sources of mortality gauged against each other, mortality incurred by parasitism emerged as a factor secondary to predation by the spiders of the High Arctic. When combined with evidence from e.g. early-successional habitats in Svalbard [95], this observation strengthens the notion of spiders as key players in the food webs of the High Arctic. From the perspective of realized population and community dynamics, the comparatively weak predation pressure exerted by shared parasitoids may thus be overridden by stronger predation by spiders.

Clearly, predation by generalist spiders may well lead to apparent competition among their prey. The evidence presented here should then not be taken as attempted proof against the occurrence of apparent competition *per se*. Instead it should be regarded as a dire warning that the mere existence of trophic interactions within a host-parasitoid sub-web may not suffice to generate tenable hypotheses about their strength, extent or the species involved in dictating population and community dynamics. To explore the consequences of spider predation, we next need to quantify how this summary force is distributed across individual herbivore species. Nonetheless, our current results suffice to suggest that the overall imprint of spider predation on lepidopteran hosts may far exceed those of the strict host-parasitoid module, and caution against far-reaching inferences drawn from any single part of a larger food web.

Interestingly, predation by birds comes across as a relatively weak force in our study area. This adds to the impression that the relative roles of different predator guilds will vary between different parts of the world (e.g. [96]). While other studies have suggested that the strength of parasitism may decrease with decreasing latitude [96], our study proposes predation by spiders as the main source of herbivore mortality in the High Arctic. Apparently, spiders may here have assumed the role of e.g. ants in the tropics [60]–[63]. Importantly, we hasten to emphasize that our comparison of mortality factors refers strictly to a single species of herbivore, as quantified by selected methods during a single year. Since estimates of mortality rates may be contingent on life-stage, weather and methods, the current comparison offers only a first step forward in understanding the functioning of Arctic food webs. Further life tables for other herbivores in the area will clearly be needed to settle the issue.

A dynamic process suggested but not satisfactorily resolved by our present study is the predation of the same prey by multiple different predator guilds. Such interguild interactions offer scope for horizontal interactions within layers of the food web. Of the parasitized larvae, some are consumed by birds and another large fraction by spiders. Thus, spiders not only compete with parasitoids for host resources, but also directly prey on parasitoids. Assuming that spiders do not discriminate between parasitized and unparasitized host individuals, they will kill a full 38% of parasitoid larvae. Yet, in other systems, parasitized prey has been found to be either more or less vulnerable to predation than unparasitized individuals ([97], [98] and references therein,[99]). If parasitized hosts proved more prone to predation in our system, then it would further enhance the role of spider predation as compared to parasitism in structuring the community of herbivores.

### Conclusions

This study critically examines the preconditions for indirect interactions as structuring an arthropod assemblage in Northeast Greenland. At the same time, it offers a first quantification of well-resolved biotic interactions within an arthropod community of the High Arctic, sheds light on the role of direct and indirect biotic interactions in a region frequently assumed to be dominated by its harsh climate and low productivity, and describes the baseline structure of a food web facing perhaps the most dramatic climate change on the planet [100]. As a perhaps more general result, our study reveals how extending the focus from a single submodule to its wider connections with the surrounding food web may change our understanding of the overall system. Only by comparing the relative strengths of different links – and potential interactions between different submodules – may we understand the forces governing the structure and dynamics of herbivorous insect communities around the world. As such, our findings caution against broad generalizations from studies of limited food web modules – and shows how concepts of quantitative overlap in host use may be fruitfully applied to multiple levels of real webs.
